# Taming of the shrewd: novel eukaryotic genes from RNA viruses

**DOI:** 10.1186/1741-7007-8-2

**Published:** 2010-01-12

**Authors:** Eugene V Koonin

**Affiliations:** 1 National Center for Biotechnology Information, National Institutes of Health, Bethesda, Maryland 20894, USA

## Abstract

Genomes of several yeast species contain integrated DNA copies of complete genomes or individual genes of non-retroviral double-strand RNA viruses as reported in a recent *BMC Biology *article by Taylor and Bruenn. The integrated virus-specific sequences are at least partially expressed and seem to evolve under pressure of purifying selection, indicating that these are functional genes. Together with similar reports on integrated copies of some animal RNA viruses, these results suggest that integration of DNA copies of non-reverse-transcribing RNA viruses might be much more common than previously thought. The integrated copies could contribute to acquired immunity to the respective viruses.

## Commentary

In a recent *BMC Biology *article Taylor and Bruenn [1] for the first time report a detailed molecular and evolutionary study of non-retroviral RNA virus genes integrated into eukaryotic genomes (hereinafter NIRV, non-retroviral integrated Rna viruses). The conclusions are no less than stunning: not only are NIRV widespread in fungi but they have become bona fide, functional genes. For retroid viruses, integration into the host genomic DNA is a regular stage of the reproduction cycle and sequences derived from retroelements comprise almost half of mammalian genomic DNA [2] and, strikingly, >75% of the genomic DNA in some plants such as maize [3]; in the more compact fungal genomes, retroelement-derived sequences are less abundant but also common [4].

So far, NIRV have been a completely different story: reliable reports of integration of DNA copies of non-retroviral RNA virus genes into host genomes can be counted on the fingers of one hand. The idea that reverse transcriptase (RT) present in eukaryotic cells could produce NIRV was first championed by Zhdanov soon after RT was discovered [5], followed by reports on integrated copies of several, diverse single-stranded RNA viruses [6,7]. However, these reports were not independently confirmed [8], with one notable exception where integrated virus-specific sequences were discovered in mice infected with lymphocytic choriomeningitis virus (LCMV), leading to the intriguing hypothesis that the integrated copies might contributed to the lifelong immunity of the survivor animals [9]. More recently, long integrated sequences, apparently derived from a novel flavivirus, were detected in the genomes of *Aedes albopictus *and *Aedes aegypti *mosquitoes [10,11]. The change of tide for NIRV seems to come from the recent work of Frank and Wolfe who surveyed the available genomes of Hemiascomycete fungi (Saccharomycotina) for sequences homologous to those of viruses and plasmids, and detected over 10 inserts derived from double-stranded (ds) RNA viruses, the dominant class of fungal RNA viruses [12], in 5 fungal species [13].

The work of Taylor and Bruenn [1] extends the results of Frank and Wolfe through a detailed characterization of NIRV derived from a specific family of dsRNA viruses (Totiviridae, typified by the L-A virus of *Saccharomyces cerevisiae*) in 5 fungal genomes, some of which carry complete copies of viral genomes whereas others possess only individual viral genes (Table 1), and adds several key findings that demonstrate the biological relevance of NIRV. First, Taylor and Bruenn unequivocally proved that there is integration of dsRNA viral genomes into the fungal genomes by polymerase chain reaction analysis of the boundaries between the integrated virus genome copy and the host DNA; the identification of the chimeric sequences leaves no doubt that the virus-specific sequences are, indeed, integral to the fungal genome. Second, it is shown that the NIRVs are completely, or partially, transcribed into polyadenylated mRNAs but no dsRNA or viral particles are formed. Third, phylogenetic analysis shows that NIRV sequences from relatively distant fungi form distinct clades in the trees of the homologous proteins of exogenous totiviruses [the capsid protein (CP) and the RNA-dependent RNA polymerase (RdRp)] suggesting that NIRVs spread by horizontal gene transfer (HGT) between fungi. Fourth, and perhaps most strikingly, a comparison of the NIRV sequences from different fungal species shows that their ratio of non-synonymous to synonymous nucleotide substitutions (Kn/Ks) is significantly less than 1 which is evidence of evolution under the pressure of purifying selection [14] (although, judging from the Ka/Ks values, that pressure is rather weak). The work of Taylor and Bruenn provides no direct clues to the mechanism of NIRV integration but there is no doubt that reverse transcription takes place, most likely, with the RT provided by Ty retroelements. Very recently, the role of endogenous retrotransposons in the LCMV integration in mice has been demonstrated directly [15].

**Table 1:** Non-retroviral integrated RNA viruses in eukaryotic genomes.

Host	Virus	Gene(s)	Transcription	References
Fungi				

*Candida parapsilosis*	Totivirus (dsRNA) related to *Saccharomyces cerevisiae *L-A (L1) virus	Capsid protein (CP)	Not tested	[1]

*Debaryomyces hansenii*	Totivirus (dsRNA) related to *S. cerevisiae *L-A (L1) virus; M2 killer virus (unclassified dsRNA virus)	CP, RdRp; apparently complete viral genome with overlapping CP and RdRp genes as in L1 virus; an extra copy of the CP gene; 4 copies of K2 toxin gene	RdRp but not Cp transcripts detected by reverse transcriptase polymerase chain reaction (RT-PCR)	[1,13]

*Penicillium marneffei*	Totivirus (dsRNA) related to *S. cerevisiae *L-A (L1) virus	CP, RdRp; apparently complete viral genome with overlapping CP and RdRp genes as in L1 virus	Not tested	[1]

*Pichia stipitus*	Totivirus (dsRNA) related to *S. cerevisiae *L-A (L1) virus	CP, RdRp; apparently complete viral genome with fused CP and RdRp genes; 3 extra copies of the CP genes	RdRp but not CP transcripts detected by RT-PCR; transcripts of both genes detected in EST database	[1,13]

*Uromyces appendiculatus*	Totivirus (dsRNA) related to *S. cerevisiae *L-A (L1) virus	CP (from EST database)	CP gene transcript detected in EST database	[1]

*Candida tropicalis*	M2 killer virus (unclassified dsRNA virus)	K2 toxin gene	Not tested	[13]

*Kluyveromyces lactis*	M2 killer virus (unclassified dsRNA virus)	K2 toxin gene (3 copies)	Not tested	[13]

*Vanderwaltozyma polyspora*	*Penicillium stoloniferum *virus F	CP	Not tested	[13]

Animals				

Mouse (*Mus musculus*)	Lymphocytic horiomeningitis virus (negative- strand ssRNA, Arenaviridae)	Glycoprotein and nucleoprotein genes (small genomic segment)	Not tested	[9,15]

Mosquitoes (*Aedes albopictus *and *Aedes aegypti*)	Flavivirus (positive- strand ssRNA) related to Cell Fusing Agent and Kamiti River virus	Non-structural protein genes (3'-terminal part of the genome)	Expressed	[10,11]

Ds, double-stranded; ss, single-stranded; RdRp, RNA-dependent RNA polymerase;

Taken together, these results indicate that the NIRV in fungal genomes encode functional proteins. So what could that function(s) be? A rather obvious possibility is protection against infection with exogenous viruses and the results of Taylor and Bruenn provide some indirect support for this hypothesis. In addition to its function as the CP protein, the totivirus CP is also an enzyme that inactivates host mRNAs by removing their 5' cap (decapping enzyme) [16]. Comparison of the NIRVs with homologous sequences of exogenous totiviruses shows that the amino acid residues required for the decapping activity are mostly replaced in the NIRVs, in accordance with the possibility that the NIRV-encoded CP is a dominant-negative inhibitor of virus encapsidation; such a function is also compatible with the duplication of the CP gene in some of the fungal genomes (Table 1; [1]). By contrast, the NIRV RdRp, at least the version from *Debaryomyces hansenii *that is available in GenBank (XM_457518), retains the catalytic residues (EVK, unpublished observations), suggesting that it could be enzymatically active. An intriguing speculation is that the NIRV RdRp could contribute to antiviral immunity by amplifying virus-specific transcripts that might protect the cell via a RNA interference mechanism. Of course, it is impossible to rule out that NIRVs and their protein products possess other, 'normal' cellular functions in addition to or even instead of their putative roles in antivirus immunity. However, the sporadic distribution of NIRV in fungal genomes and, even more importantly, their likely spread via HGT appear to be better compatible with the immunity hypothesis.

The possibility that fungal NIRVs provide resistance against exogenous viruses puts them into the context of the burgeoning research area of 'integration-based immunity'. A novel form of acquired, adaptive antiviral immunity that has been recently discovered in bacteria and archaea is mediated by the so-called CRISPR (clustered regularly interspaced short palindromic repeats) system. This system functions by integrating small segments of virus (bacteriophage) DNA into specific loci in the host genome and utilizing them to abrogate the cognate virus replication, most probably via an RNA interference-like mechanism [17,18]. The CRISPR system functioning involves intricate, specialized molecular mechanisms mediated by the Cas (CRISPR-associated) genes [19]. The NIRV-mediated immunity, if a real phenomenon, seems to rely on more generic mechanism of regular gene expression. The principle of acquired immunity, nevertheless, would remain the same: capture of virus-specific sequences and their subsequent use against the virus. Integration-based immunity might be even more widespread: recent reports reveal multiple inserts of pararetrovirus sequences in a genome of grapevine, a plant that appears to be resistant to active viruses of this group [20] and inserts of short fragments of both RNA and DNA viral genomes in insects and crustaceans [21]. A tempting general speculation is that integration of virus-specific sequences into the host genome resulting in acquired immunity is a ubiquitous route of virus-host interaction that is manifest through a plethora of specific mechanisms.

The second important message of the NIRV story is the apparent relatively recent HGT between fungi [1]. The prevalence and roles of HGT in eukaryotic evolution is a rather controversial matter, with relatively few iron-clad cases [22]. However, the observations on NIRVs, along with the recent demonstration of multiple acquisitions of bacterial genes by fungi [23] shows that, although HGT is probably much less pervasive in eukaryotes than it is in prokaryotes, sequencing of multiple, diverse eukaryotic genomes reveals substantial lateral gene traffic. With the advent of the new generation of sequencing methods, genomics is rapidly expanding, and there is no doubt that we will be in for many more surprises.

## Abbreviations

CP: capsid protein; CRISPR: cluster regularly interspersed short palindromic repeats; ds: double stranded; HGT: horizontal gene transfer; NIRV: non-retroviral integrated Rna viruses; LCMV: lymphocytic choriomeningitis virus; RdRp: RNA-dependant RNA polymerase; RT: reverse transcriptase.
